# *catena*-Poly[sodium-di-μ-aqua-sodium-bis[μ-2,2,2-trichloro-*N*-(dimorpholinophosphoryl)acetamide]]

**DOI:** 10.1107/S1600536810009670

**Published:** 2010-03-20

**Authors:** Olena O. Litsis, Vladimir A. Ovchynnikov, Tetyana Yu. Sliva, Irina S. Konovalova, Vladimir M. Amirkhanov

**Affiliations:** a Kyiv National Taras Shevchenko University, Department of Chemistry, Volodymyrska str. 64, 01601 Kyiv, Ukraine; b STC "Institute for Single Crystals", National Academy of Science of Ukraine, Lenina ave. 60, 61001, Khar’kov, Ukraine

## Abstract

The title compound, [Na_2_(C_10_H_16_Cl_3_N_3_O_4_P)_2_(H_2_O)_2_]_*n*_, can be considered as a two-dimensional coordination polymer in which one-dimensional chains are connected to each other by inter­molecular C—H⋯O hydrogen bonds involving the water mol­ecules. The Na^I^ ion is five-coordinated in a distorted trigonal-bipyramidal geometry. The connection between the two Na^I^ ions is facilitated by the two μ-O atoms of the carbonyl group of the 2,2,2-trichloro-*N*-(dimorpholino­phosphor­yl)acetamide (CAPh) ligand. A bridging coordination of the CAPh ligand *via* the carbonyl O atom is observed for the first time. The bridging water mol­ecules form inter­molecular O—H⋯O hydrogen bonds with the O atoms of the morpholine rings and the phosphoryl groups of neighboring CAPh mol­ecules.

## Related literature

For the pharmacological and biological properties of carbacyl­amido­phosphate (CAPh) derivatives, see: Barak *et al.* (2000 ▶); Grimes *et al.* (2008 ▶); Adams *et al.* (2002 ▶); For structural analogues of phospho­rylated carbacyl­amides and their coordination properties, see: Amirkhanov *et al.* (1996 ▶); Rebrova *et al.* (1982 ▶); Gubina *et al.* (1999 ▶); Ovchinnikov *et al.* (2001 ▶); Gholivand & Shariatinia (2006 ▶); Trush *et al.* (2005 ▶); Zhang *et al.* (1992 ▶). For details of the synthesis, see: Kirsanov & Derkach (1956 ▶). For the synthesis of the 2,2,2-trichloro-*N*-(dimorpholinophosphoryl)acetamide (H*L*) ligand, see: Ovchyn­nikov *et al.* (1998 ▶). For coordination compounds of H*L*, see: Ovchynnikov *et al.* (2000 ▶); Trush *et al.* (2002 ▶, 2003 ▶). For the trigonality index τ, see: Addison *et al.* (1984 ▶).
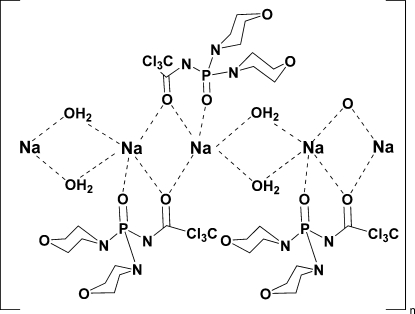


## Experimental

### 

#### Crystal data

[Na_2_(C_10_H_16_Cl_3_N_3_O_4_P)_2_(H_2_O)_2_]
*M* *_r_* = 841.17
Triclinic, 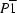

*a* = 7.522 (5) Å
*b* = 10.329 (4) Å
*c* = 12.451 (5) Å
α = 84.17 (4)°
β = 80.89 (4)°
γ = 70.16 (5)°
*V* = 897.3 (8) Å^3^
*Z* = 1
Mo *K*α radiation
μ = 0.65 mm^−1^
*T* = 294 K
0.40 × 0.30 × 0.20 mm

#### Data collection

Oxford Diffraction Xcalibur3 diffractometer
Absorption correction: multi-scan (*CrysAlis RED*; Oxford Diffraction, 2006 ▶) *T*_min_ = 0.782, *T*_max_ = 0.938
10258 measured reflections
5137 independent reflections
3339 reflections with *I* > 2σ(*I*)
*R*_int_ = 0.027

#### Refinement

*R*[*F* ^2^ > 2σ(*F* ^2^)] = 0.046
*wR*(*F* ^2^) = 0.121
*S* = 0.95
5137 reflections
236 parameters
6 restraints
H-atom parameters constrained
Δρ_max_ = 0.44 e Å^−3^
Δρ_min_ = −0.55 e Å^−3^

### 

Data collection: *CrysAlis CCD* (Oxford Diffraction, 2006 ▶); cell refinement: *CrysAlis RED* (Oxford Diffraction, 2006 ▶); data reduction: *CrysAlis RED*; program(s) used to solve structure: *SHELXTL* (Sheldrick, 2008 ▶); program(s) used to refine structure: *SHELXTL*; molecular graphics: *XP* in *SHELXTL*; software used to prepare material for publication: *SHELXTL*.

## Supplementary Material

Crystal structure: contains datablocks I, global. DOI: 10.1107/S1600536810009670/jh2135sup1.cif

Structure factors: contains datablocks I. DOI: 10.1107/S1600536810009670/jh2135Isup2.hkl

Additional supplementary materials:  crystallographic information; 3D view; checkCIF report

## Figures and Tables

**Table 1:** Hydrogen-bond geometry (Å, °)

*D*—H⋯*A*	*D*—H	H⋯*A*	*D*⋯*A*	*D*—H⋯*A*
C3—H3*B*⋯O1^i^	0.97	2.59	3.443 (4)	147
O1*W*—H1*WA*⋯O3^ii^	0.98	1.77	2.716 (3)	163
O1*W*—H1*WB*⋯O2^iii^	0.98	2.00	2.917 (3)	155

Symmetry codes: (i) 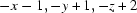; (ii) 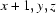; (iii) 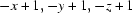.

**Fig. 1.**
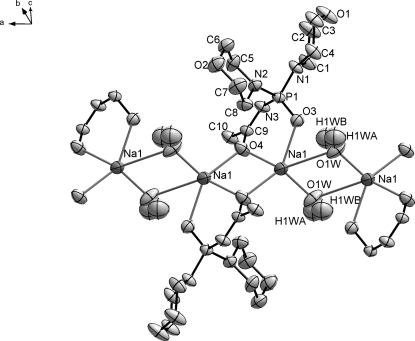
A portion of polymeric chain of the title compound, showing the 30% probability displacement ellipsoids and atomic numbering [symmetry codes: ]. H atoms of L- and Cl atoms of trychlormethyl groups have been omitted for clarity.

**Fig. 2.**
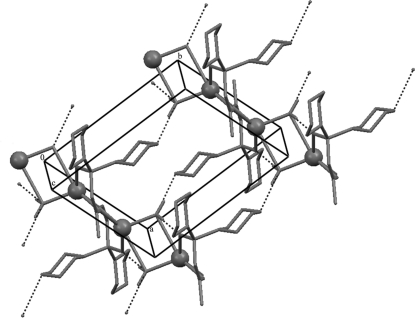
A schematic view of packing diagram from [Na(L)(H2O)]n (projection along the y direction). H atoms and Cl atoms of trychlormethyl groups have been omitted for clarity.

## supplementary crystallographic information

### Comment

Carbacylamidophosphate compounds have been attracting substantial interest and are widely used to date. These compounds have been employed in pharmacology as potential novel antibacterial agents and prodrugs (Adams *et al.*, 2002, Kimberly D. Grimes *et al.*, 2008); some carbacylamidophosphates are effective pesticides (Barak *et al.*, 2000). The ability of carbacylamidophosphates to form stable complexes both with transition and non-transition metals via their =P(O)N(H)C(O)- moiety has been investigated extensively by Amirkhanov *et al.*, 1996, Trush *et al.*, 2005, Ovchinnikov *et al.*, 2001, Gholivand *et al.*, 2006, Wenjun Zhang *et al.*, 1992. This paper is devoted to the crystal structure of the sodium salt of 2,2,2-trichloro-*N*-(dimorpholin-4-yl-phosphoryl)acetamide (HL) NaL and the first fact of bridging coordination of CAPh ligand via carbonyl oxygen. Coordination compounds of 4f-metal ions with HL have been reported earlier (Ovchynnikov *et al.*, 2000, Trush *et al.*, 2002, Trush *et al.*, 2003).

The molecular structure of the title compound is shown in Fig. 1. The structure is build up of [C_10_H_18_Cl_3_N_3_NaO_5_P]n chains along [001]. The polymeric chain contains Na atoms, which are five-coordinated by three O atoms of 2 HL molecules and two O atoms of water. Each CAPh ligand links Na^+^ centers via its phosphoryl and carbonyl groups in a chelating manner. Oxygen atom of carbonyl group is a bridging atom between two sodium ions. The value of the trigonality index τ (τ = (β-α)/60, where α and β are the largest coordination angles) (Addison *et al.*, 1984) is 0,049 for Na(1) [α = O(4)—Na(1)—O(1 W) = 140,69°, β = O(3)—Na(1)—O(4) = 143,63°]. It indicates that sodium (I) ion is in a distorted trigonal bipyramidal coordination geometry. One of the equatorial distances is significantly longer [Na(1)—O(1 W) = 3,022 Å] than all other Na—O distances, which are almost equivalent. The values of the O—Na—O angles also reveal the strong deviation of the sodium (I) atom environment from the ideal trigonal- bipyramidal geometry. The P=O and C=O distances in the chelate ring and P—N distances in the morpholine substituents of L^-^ in the sodium salt are longer than in the free ligand (i. e. uncoordinated) (Table 1). But the P—N_amide_ distance is shortened upon coordination, indicating the presence of π-conjugation in the coordinated anion. Carbonyl group oxygen forms two types of bonds with Na: intrachelating bond O—Na is some longer, than bond with other Na atom. The bridging water molecules are involved in hydrogen bonding interactions (Table 1). Intramolecular hydrogen bonds stabilize the two-dimensional structure of the title compound. They are oriented towards the neighboring oxygen atom O(2) of the morpholine rings. The other H atom of the water molecule makes a strong intermolecular H bond to O(3) of P=O group of neighboring L^-^ molecule. The intermolecular hydrogen bonds are arranged in inversion symmetric pairs that connect molecules along the c-axis leading to strongly hydrogen bonded strings of the molecules along that axis (Figure 2).

### Experimental

The synthesis of HL was carried out according to the method described early (Ovchynnikov *et al.*, 1998).

HL (0,38 g, 1 mmol) was dissolved in methanol (10 ml) and added to 10 ml of sodium methoxide (0,023 g, 1 mmol of Na in methanol). After 20 min the solution was evaporated and the residue was dissolved in water. The resulting clear solution was left at ambient temperature for crystallization in air. The crystals were separated by filtration after 48 h and dried in air. Yield: 95-98%. IR (KBr pellet, cm^-1^): 1605 (s, CO), 1344 (Amide II), 1152 (s, PO).

### Crystal data

**Table:**

[Na_2_(C_10_H_16_Cl_3_N_3_O_4_P)_2_(H_2_O)_2_]	*Z* = 1
*M**_r_* = 841.17	*F*(000) = 432
Triclinic, *P*1	*D*_x_ = 1.557 Mg m^−^^3^
Hall symbol: -P 1	Mo *K*α radiation, λ = 0.71073 Å
*a* = 7.522 (5) Å	Cell parameters from 2305 reflections
*b* = 10.329 (4) Å	θ = 2.9–32.1°
*c* = 12.451 (5) Å	µ = 0.65 mm^−^^1^
α = 84.17 (4)°	*T* = 294 K
β = 80.89 (4)°	Block, colourless
γ = 70.16 (5)°	0.40 × 0.30 × 0.20 mm
*V* = 897.3 (8) Å^3^	

### Data collection

**Table:**

Oxford Diffraction Xcalibur3 diffractometer	5137 independent reflections
Radiation source: Enhance (Mo) X-ray Source	3339 reflections with *I* > 2σ(*I*)
graphite	*R*_int_ = 0.027
Detector resolution: 16.1827 pixels mm^-1^	θ_max_ = 30.0°, θ_min_ = 3.0°
ω scans	*h* = −9→10
Absorption correction: multi-scan (*CrysAlis RED*; Oxford Diffraction, 2006)	*k* = −14→14
*T*_min_ = 0.782, *T*_max_ = 0.938	*l* = −17→17
10258 measured reflections	

### Refinement

**Table:**

Refinement on *F*^2^	Primary atom site location: structure-invariant direct methods
Least-squares matrix: full	Secondary atom site location: difference Fourier map
*R*[*F*^2^ > 2σ(*F*^2^)] = 0.046	Hydrogen site location: difference Fourier map
*wR*(*F*^2^) = 0.121	H-atom parameters constrained
*S* = 0.95	*w* = 1/[σ^2^(*F*_o_^2^) + (0.0695*P*)^2^] where *P* = (*F*_o_^2^ + 2*F*_c_^2^)/3
5137 reflections	(Δ/σ)_max_ < 0.001
236 parameters	Δρ_max_ = 0.44 e Å^−^^3^
6 restraints	Δρ_min_ = −0.55 e Å^−^^3^

### Special details

**Table:**

Geometry. All esds (except the esd in the dihedral angle between two l.s. planes) are estimated using the full covariance matrix. The cell esds are taken into account individually in the estimation of esds in distances, angles and torsion angles; correlations between esds in cell parameters are only used when they are defined by crystal symmetry. An approximate (isotropic) treatment of cell esds is used for estimating esds involving l.s. planes.
Refinement. Refinement of *F*^2^ against ALL reflections. The weighted *R*-factor wR and goodness of fit *S* are based on *F*^2^, conventional *R*-factors *R* are based on *F*, with *F* set to zero for negative *F*^2^. The threshold expression of *F*^2^ > σ(*F*^2^) is used only for calculating *R*-factors(gt) etc. and is not relevant to the choice of reflections for refinement. *R*-factors based on *F*^2^ are statistically about twice as large as those based on *F*, and *R*- factors based on ALL data will be even larger.

### Fractional atomic coordinates and isotropic or equivalent isotropic displacement parameters (Å^2^)

**Table:**

	*x*	*y*	*z*	*U*_iso_*/*U*_eq_	Occ. (<1)
Na1	0.74853 (12)	−0.02122 (8)	0.49141 (6)	0.0462 (2)	
P1	0.14168 (7)	0.19927 (5)	0.73292 (4)	0.03306 (13)	
Cl1	0.7919 (6)	−0.0374 (6)	0.7663 (5)	0.0975 (19)	0.30
Cl2	0.5558 (14)	−0.1440 (9)	0.9126 (2)	0.082 (3)	0.30
Cl3	0.6925 (9)	−0.2632 (4)	0.7073 (5)	0.109 (2)	0.30
Cl1A	0.8282 (3)	−0.0794 (3)	0.7362 (2)	0.1334 (12)	0.70
Cl2A	0.5706 (5)	−0.1301 (4)	0.91551 (8)	0.0708 (8)	0.70
Cl3A	0.6346 (6)	−0.26558 (18)	0.7220 (3)	0.1413 (14)	0.70
N1	−0.0076 (2)	0.25733 (16)	0.84197 (13)	0.0387 (4)	
N2	0.2136 (2)	0.33001 (16)	0.68794 (12)	0.0376 (3)	
N3	0.3105 (2)	0.07308 (16)	0.78548 (13)	0.0398 (4)	
O1	−0.2764 (2)	0.36756 (19)	1.02222 (14)	0.0712 (5)	
O2	0.3238 (3)	0.55916 (17)	0.61132 (14)	0.0605 (4)	
O3	0.05740 (19)	0.16343 (14)	0.64384 (11)	0.0436 (3)	
O4	0.4863 (2)	0.00857 (16)	0.61885 (11)	0.0492 (4)	
C1	−0.0371 (3)	0.1708 (2)	0.93747 (19)	0.0533 (5)	
H1B	0.0821	0.0987	0.9481	0.064*	
H1A	−0.1285	0.1274	0.9268	0.064*	
C2	−0.1092 (4)	0.2545 (3)	1.03586 (19)	0.0689 (7)	
H2A	−0.1360	0.1962	1.0980	0.083*	
H2B	−0.0108	0.2885	1.0511	0.083*	
C3	−0.2430 (4)	0.4527 (2)	0.9306 (2)	0.0621 (6)	
H3A	−0.1457	0.4902	0.9421	0.074*	
H3B	−0.3590	0.5292	0.9222	0.074*	
C4	−0.1799 (3)	0.3747 (2)	0.82922 (18)	0.0517 (5)	
H4B	−0.2802	0.3423	0.8146	0.062*	
H4A	−0.1544	0.4347	0.7680	0.062*	
C5	0.3079 (4)	0.3877 (2)	0.75538 (18)	0.0520 (5)	
H5B	0.4445	0.3403	0.7443	0.062*	
H5A	0.2618	0.3753	0.8317	0.062*	
C6	0.2665 (4)	0.5365 (3)	0.7248 (2)	0.0583 (6)	
H6A	0.1309	0.5843	0.7421	0.070*	
H6B	0.3332	0.5744	0.7671	0.070*	
C7	0.2333 (4)	0.5012 (2)	0.54594 (19)	0.0577 (6)	
H7A	0.2777	0.5158	0.4696	0.069*	
H7B	0.0966	0.5477	0.5578	0.069*	
C8	0.2748 (3)	0.3510 (2)	0.57246 (16)	0.0462 (5)	
H8B	0.2074	0.3148	0.5293	0.055*	
H8A	0.4103	0.3027	0.5556	0.055*	
C9	0.4531 (3)	0.00130 (18)	0.71992 (15)	0.0344 (4)	
C10	0.61717 (19)	−0.11305 (14)	0.77559 (8)	0.0466 (5)	
O1W	0.8336 (3)	0.16719 (17)	0.49163 (14)	0.0647 (5)	
H1WA	0.8908	0.1732	0.5556	0.097*	
H1WB	0.7821	0.2673	0.4784	0.097*	

### Atomic displacement parameters (Å^2^)

**Table:**

	*U*^11^	*U*^22^	*U*^33^	*U*^12^	*U*^13^	*U*^23^
Na1	0.0504 (5)	0.0472 (5)	0.0453 (4)	−0.0219 (4)	0.0012 (4)	−0.0133 (4)
P1	0.0316 (2)	0.0281 (2)	0.0357 (2)	−0.00463 (17)	−0.00432 (19)	−0.00264 (18)
Cl1	0.064 (3)	0.116 (3)	0.139 (5)	−0.054 (3)	−0.054 (3)	0.027 (3)
Cl2	0.101 (5)	0.052 (2)	0.050 (3)	0.014 (2)	0.008 (3)	0.025 (2)
Cl3	0.134 (3)	0.065 (3)	0.057 (2)	0.067 (2)	−0.013 (2)	−0.0271 (19)
Cl1A	0.0365 (6)	0.226 (3)	0.1052 (14)	−0.0258 (11)	−0.0134 (7)	0.0845 (18)
Cl2A	0.0624 (10)	0.0851 (18)	0.0385 (10)	0.0109 (10)	−0.0100 (8)	−0.0020 (9)
Cl3A	0.274 (4)	0.0300 (8)	0.0826 (14)	−0.0019 (12)	−0.0180 (19)	−0.0110 (7)
N1	0.0328 (8)	0.0308 (8)	0.0405 (8)	0.0016 (6)	0.0014 (7)	0.0008 (7)
N2	0.0426 (8)	0.0367 (8)	0.0346 (8)	−0.0143 (7)	−0.0067 (7)	−0.0013 (7)
N3	0.0375 (8)	0.0341 (8)	0.0377 (8)	0.0006 (6)	−0.0022 (7)	−0.0033 (7)
O1	0.0557 (10)	0.0682 (11)	0.0529 (10)	0.0173 (8)	0.0118 (8)	0.0019 (9)
O2	0.0806 (12)	0.0514 (9)	0.0615 (10)	−0.0379 (9)	−0.0111 (9)	0.0026 (8)
O3	0.0414 (7)	0.0408 (7)	0.0504 (8)	−0.0124 (6)	−0.0115 (7)	−0.0057 (6)
O4	0.0415 (7)	0.0600 (9)	0.0353 (7)	−0.0027 (7)	−0.0040 (6)	−0.0034 (6)
C1	0.0492 (12)	0.0393 (11)	0.0546 (13)	−0.0023 (9)	0.0071 (11)	0.0079 (10)
C2	0.0613 (15)	0.0683 (17)	0.0442 (12)	0.0153 (12)	0.0022 (12)	0.0038 (12)
C3	0.0486 (12)	0.0448 (13)	0.0688 (16)	0.0101 (10)	0.0054 (12)	−0.0041 (11)
C4	0.0375 (10)	0.0479 (12)	0.0509 (12)	0.0067 (9)	−0.0033 (9)	0.0057 (10)
C5	0.0600 (13)	0.0602 (14)	0.0483 (12)	−0.0318 (12)	−0.0184 (11)	0.0008 (10)
C6	0.0734 (16)	0.0563 (14)	0.0560 (13)	−0.0320 (13)	−0.0115 (13)	−0.0104 (11)
C7	0.0838 (17)	0.0493 (13)	0.0477 (12)	−0.0317 (13)	−0.0145 (12)	0.0066 (10)
C8	0.0576 (12)	0.0406 (11)	0.0377 (10)	−0.0158 (10)	0.0021 (9)	−0.0047 (9)
C9	0.0346 (9)	0.0284 (8)	0.0369 (9)	−0.0060 (7)	−0.0045 (8)	−0.0019 (7)
C10	0.0432 (10)	0.0420 (11)	0.0400 (10)	0.0017 (9)	−0.0003 (9)	0.0005 (9)
O1W	0.0899 (12)	0.0424 (9)	0.0748 (11)	−0.0292 (9)	−0.0365 (10)	0.0044 (8)

### Geometric parameters (Å, °)

**Table:**

Na1—O1W	2.2458 (19)	O3—Na1^i^	2.322 (2)
Na1—O4	2.280 (2)	O4—C9	1.243 (2)
Na1—O3^i^	2.322 (2)	O4—Na1^i^	2.366 (2)
Na1—O4^i^	2.366 (2)	C1—C2	1.493 (3)
Na1—Cl1A	3.158 (3)	C1—H1B	0.9700
Na1—P1^i^	3.3388 (19)	C1—H1A	0.9700
P1—O3	1.4949 (15)	C2—H2A	0.9700
P1—O3	1.4949 (15)	C2—H2B	0.9700
P1—N2	1.6358 (18)	C3—C4	1.492 (4)
P1—N1	1.6401 (19)	C3—H3A	0.9700
P1—N3	1.645 (2)	C3—H3B	0.9700
P1—Na1^i^	3.3388 (19)	C4—H4B	0.9700
Cl1—C10	1.7264 (14)	C4—H4A	0.9700
Cl2—C10	1.7219 (13)	C5—C6	1.483 (3)
Cl3—C10	1.7222 (14)	C5—H5B	0.9700
Cl1A—C10	1.7246 (15)	C5—H5A	0.9700
Cl2A—C10	1.7248 (12)	C6—H6A	0.9700
Cl3A—C10	1.7290 (13)	C6—H6B	0.9700
N1—C1	1.448 (3)	C7—C8	1.488 (3)
N1—C4	1.461 (3)	C7—H7A	0.9700
N2—C8	1.456 (2)	C7—H7B	0.9700
N2—C5	1.463 (3)	C8—H8B	0.9700
N3—C9	1.293 (3)	C8—H8A	0.9700
O1—C3	1.412 (3)	C9—C10	1.586 (3)
O1—C2	1.416 (3)	O1W—H1WA	0.9800
O2—C7	1.427 (3)	O1W—H1WB	0.9800
O2—C6	1.430 (3)		
			
O1W—Na1—O4	106.11 (9)	O1—C2—H2B	109.2
O1W—Na1—O3^i^	110.04 (8)	C1—C2—H2B	109.2
O4—Na1—O3^i^	143.65 (7)	H2A—C2—H2B	107.9
O1W—Na1—O4^i^	117.21 (8)	O1—C3—C4	111.4 (2)
O4—Na1—O4^i^	78.92 (7)	O1—C3—H3A	109.4
O3^i^—Na1—O4^i^	81.39 (7)	C4—C3—H3A	109.4
O1W—Na1—Cl1A	86.78 (8)	O1—C3—H3B	109.4
O4—Na1—Cl1A	64.22 (7)	C4—C3—H3B	109.4
O3^i^—Na1—Cl1A	121.01 (9)	H3A—C3—H3B	108.0
O4^i^—Na1—Cl1A	140.84 (6)	N1—C4—C3	109.77 (19)
O1W—Na1—P1^i^	119.98 (7)	N1—C4—H4B	109.7
O4—Na1—P1^i^	127.30 (6)	C3—C4—H4B	109.7
O3^i^—Na1—P1^i^	22.70 (4)	N1—C4—H4A	109.7
O4^i^—Na1—P1^i^	58.77 (6)	C3—C4—H4A	109.7
Cl1A—Na1—P1^i^	137.07 (7)	H4B—C4—H4A	108.2
O1W—Na1—Na1^i^	118.55 (8)	N2—C5—C6	108.99 (19)
O4—Na1—Na1^i^	40.33 (5)	N2—C5—H5B	109.9
O3^i^—Na1—Na1^i^	114.40 (6)	C6—C5—H5B	109.9
O4^i^—Na1—Na1^i^	38.58 (5)	N2—C5—H5A	109.9
Cl1A—Na1—Na1^i^	103.59 (6)	C6—C5—H5A	109.9
P1^i^—Na1—Na1^i^	92.48 (5)	H5B—C5—H5A	108.3
O1W—Na1—Na1^ii^	48.58 (7)	O2—C6—C5	111.5 (2)
O4—Na1—Na1^ii^	130.58 (6)	O2—C6—H6A	109.3
O3^i^—Na1—Na1^ii^	78.91 (6)	C5—C6—H6A	109.3
O4^i^—Na1—Na1^ii^	147.34 (6)	O2—C6—H6B	109.3
Cl1A—Na1—Na1^ii^	71.78 (5)	C5—C6—H6B	109.3
P1^i^—Na1—Na1^ii^	100.05 (5)	H6A—C6—H6B	108.0
Na1^i^—Na1—Na1^ii^	165.63 (5)	O2—C7—C8	111.4 (2)
O3—P1—N2	107.86 (9)	O2—C7—H7A	109.3
O3—P1—N2	107.86 (9)	C8—C7—H7A	109.3
O3—P1—N1	115.65 (9)	O2—C7—H7B	109.3
O3—P1—N1	115.65 (9)	C8—C7—H7B	109.3
N2—P1—N1	102.81 (9)	H7A—C7—H7B	108.0
O3—P1—N3	116.42 (9)	N2—C8—C7	108.85 (18)
O3—P1—N3	116.42 (9)	N2—C8—H8B	109.9
N2—P1—N3	111.58 (10)	C7—C8—H8B	109.9
N1—P1—N3	101.70 (9)	N2—C8—H8A	109.9
N2—P1—Na1^i^	100.79 (7)	C7—C8—H8A	109.9
N1—P1—Na1^i^	149.19 (7)	H8B—C8—H8A	108.3
N3—P1—Na1^i^	87.59 (8)	O4—C9—N3	130.61 (18)
C10—Cl1A—Na1	91.55 (11)	O4—C9—C10	113.42 (15)
C1—N1—C4	111.08 (17)	N3—C9—C10	115.95 (15)
C1—N1—P1	123.56 (14)	C9—C10—Cl2	113.7 (3)
C4—N1—P1	118.63 (14)	C9—C10—Cl3	110.5 (2)
C8—N2—C5	111.34 (16)	Cl2—C10—Cl3	110.9 (4)
C8—N2—P1	120.92 (13)	C9—C10—Cl1A	108.77 (14)
C5—N2—P1	121.27 (14)	Cl2—C10—Cl1A	117.4 (4)
C9—N3—P1	118.23 (14)	Cl3—C10—Cl1A	93.9 (3)
C3—O1—C2	110.45 (18)	C9—C10—Cl2A	114.42 (16)
C7—O2—C6	111.22 (16)	Cl3—C10—Cl2A	116.2 (3)
P1—O3—Na1^i^	120.47 (9)	Cl1A—C10—Cl2A	111.12 (18)
C9—O4—Na1	136.68 (13)	C9—C10—Cl1	102.9 (2)
C9—O4—Na1^i^	121.40 (13)	Cl2—C10—Cl1	106.0 (5)
Na1—O4—Na1^i^	101.08 (7)	Cl3—C10—Cl1	112.7 (3)
N1—C1—C2	110.33 (19)	Cl2A—C10—Cl1	98.9 (3)
N1—C1—H1B	109.6	C9—C10—Cl3A	104.82 (15)
C2—C1—H1B	109.6	Cl2—C10—Cl3A	102.2 (4)
N1—C1—H1A	109.6	Cl1A—C10—Cl3A	109.0 (2)
C2—C1—H1A	109.6	Cl2A—C10—Cl3A	108.5 (2)
H1B—C1—H1A	108.1	Cl1—C10—Cl3A	127.7 (3)
O1—C2—C1	112.3 (2)	Na1—O1W—H1WA	115.1
O1—C2—H2A	109.2	Na1—O1W—H1WB	139.9
C1—C2—H2A	109.2	H1WA—O1W—H1WB	94.1
			
O1W—Na1—Cl1A—C10	133.78 (14)	O1W—Na1—O4—Na1^i^	115.37 (8)
O4—Na1—Cl1A—C10	24.16 (11)	O3^i^—Na1—O4—Na1^i^	−58.44 (12)
O3^i^—Na1—Cl1A—C10	−114.71 (13)	O4^i^—Na1—O4—Na1^i^	0.0
O4^i^—Na1—Cl1A—C10	2.8 (2)	Cl1A—Na1—O4—Na1^i^	−166.44 (9)
P1^i^—Na1—Cl1A—C10	−93.81 (14)	P1^i^—Na1—O4—Na1^i^	−35.57 (8)
Na1^i^—Na1—Cl1A—C10	15.17 (14)	Na1^ii^—Na1—O4—Na1^i^	164.12 (7)
Na1^ii^—Na1—Cl1A—C10	−178.99 (14)	C4—N1—C1—C2	−53.9 (3)
O3—P1—N1—C1	94.71 (18)	P1—N1—C1—C2	155.41 (17)
O3—P1—N1—C1	94.71 (18)	C3—O1—C2—C1	−57.4 (3)
N2—P1—N1—C1	−148.03 (17)	N1—C1—C2—O1	55.1 (3)
N3—P1—N1—C1	−32.41 (19)	C2—O1—C3—C4	58.7 (3)
Na1^i^—P1—N1—C1	72.9 (2)	C1—N1—C4—C3	55.3 (3)
O3—P1—N1—C4	−53.94 (18)	P1—N1—C4—C3	−152.41 (17)
O3—P1—N1—C4	−53.94 (18)	O1—C3—C4—N1	−57.7 (3)
N2—P1—N1—C4	63.32 (17)	C8—N2—C5—C6	57.6 (3)
N3—P1—N1—C4	178.94 (15)	P1—N2—C5—C6	−150.46 (17)
Na1^i^—P1—N1—C4	−75.8 (2)	C7—O2—C6—C5	57.2 (3)
O3—P1—N2—C8	−29.02 (18)	N2—C5—C6—O2	−56.6 (3)
O3—P1—N2—C8	−29.02 (18)	C6—O2—C7—C8	−57.4 (3)
N1—P1—N2—C8	−151.68 (16)	C5—N2—C8—C7	−57.7 (2)
N3—P1—N2—C8	100.04 (17)	P1—N2—C8—C7	150.24 (17)
Na1^i^—P1—N2—C8	8.36 (16)	O2—C7—C8—N2	57.1 (3)
O3—P1—N2—C5	−178.34 (16)	Na1—O4—C9—N3	144.80 (18)
O3—P1—N2—C5	−178.34 (16)	Na1^i^—O4—C9—N3	−47.9 (3)
N1—P1—N2—C5	59.00 (19)	Na1—O4—C9—C10	−33.5 (3)
N3—P1—N2—C5	−49.28 (19)	Na1^i^—O4—C9—C10	133.83 (12)
Na1^i^—P1—N2—C5	−140.96 (16)	P1—N3—C9—O4	−1.7 (3)
O3—P1—N3—C9	52.00 (18)	P1—N3—C9—C10	176.53 (10)
O3—P1—N3—C9	52.00 (18)	O4—C9—C10—Cl2	−169.7 (4)
N2—P1—N3—C9	−72.38 (17)	N3—C9—C10—Cl2	11.8 (4)
N1—P1—N3—C9	178.63 (15)	O4—C9—C10—Cl3	−44.2 (3)
Na1^i^—P1—N3—C9	28.27 (15)	N3—C9—C10—Cl3	137.3 (3)
N2—P1—O3—O3	0.00 (17)	O4—C9—C10—Cl1A	57.5 (2)
N1—P1—O3—O3	0.00 (13)	N3—C9—C10—Cl1A	−121.00 (19)
N3—P1—O3—O3	0.00 (14)	O4—C9—C10—Cl2A	−177.6 (2)
Na1^i^—P1—O3—O3	0.00 (14)	N3—C9—C10—Cl2A	3.9 (3)
O3—P1—O3—Na1^i^	0(50)	O4—C9—C10—Cl1	76.3 (3)
N2—P1—O3—Na1^i^	84.15 (12)	N3—C9—C10—Cl1	−102.3 (3)
N1—P1—O3—Na1^i^	−161.45 (8)	O4—C9—C10—Cl3A	−58.9 (2)
N3—P1—O3—Na1^i^	−42.12 (12)	N3—C9—C10—Cl3A	122.6 (2)
O1W—Na1—O4—C9	−75.7 (2)	Na1—Cl1A—C10—C9	−44.71 (14)
O3^i^—Na1—O4—C9	110.5 (2)	Na1—Cl1A—C10—Cl2	−175.6 (3)
O4^i^—Na1—O4—C9	169.0 (2)	Na1—Cl1A—C10—Cl3	68.4 (2)
Cl1A—Na1—O4—C9	2.54 (19)	Na1—Cl1A—C10—Cl2A	−171.53 (16)
P1^i^—Na1—O4—C9	133.41 (18)	Na1—Cl1A—C10—Cl1	−119.3 (9)
Na1^i^—Na1—O4—C9	169.0 (2)	Na1—Cl1A—C10—Cl3A	69.00 (16)
Na1^ii^—Na1—O4—C9	−26.9 (2)		

Symmetry codes: (i) −*x*+1, −*y*, −*z*+1;  (ii) −*x*+2, −*y*, −*z*+1.

### Hydrogen-bond geometry (Å, °)

**Table:**

*D*—H···*A*	*D*—H	H···*A*	*D*···*A*	*D*—H···*A*
C3—H3B···O1^iii^	0.97	2.59	3.443 (4)	147
O1W—H1WA···O3^iv^	0.98	1.77	2.716 (3)	163
O1W—H1WB···O2^v^	0.98	2.00	2.917 (3)	155

Symmetry codes: (iii) −*x*−1, −*y*+1, −*z*+2; (iv) *x*+1, *y*, *z*; (v) −*x*+1, −*y*+1, −*z*+1.
